# The Evolutionary Route of in vitro Human Spermatogenesis: What is the Next Destination?

**DOI:** 10.1007/s12015-024-10726-2

**Published:** 2024-04-29

**Authors:** Merve Gizer, Selin Önen, Petek Korkusuz

**Affiliations:** 1 Department of Stem Cell Sciences, Graduate School of Health Sciences, Hacettepe University, 06100 Ankara, Turkey; 2 METU MEMS Center, 06530 Ankara, Turkey; 3 Department of Histology and Embryology, Faculty of Medicine, Hacettepe University, Sihhiye, 06100 Ankara, Turkey

**Keywords:** Induced Pluripotent Stem Cells, Spermatogonial Stem Cells, In vitro Spermatogenesis, Male Factor Infertility

## Abstract

Malfunction in spermatogenesis due to genetic diseases, trauma, congenital disorders or gonadotoxic treatments results in infertility in approximately 7% of males. The behavior of spermatogonial stem cells (SSCs) within three-dimensional, multifactorial, and dynamic microenvironment implicates a niche that serves as a repository for fertility, since can serve as a source of mature and functional male germ cells. Current protocols enable reprogramming of mature somatic cells into induced pluripotent stem cells (iPSCs) and their limited differentiation to SSCs within the range of 0–5%. However, the resulting human iPSC-derived haploid spermatogenic germ cell yield in terms of number and functionality is currently insufficient for transfer to infertility clinic as a therapeutic tool. In this article, we reviewed the evolution of experimental culture platforms and introduced a novel iPSCs-based approach for in vitro spermatogenesis based on a niche perspective bearing cellular, chemical, and physical factors that provide the complex arrangement of testicular seminiferous tubules embedded within a vascularized stroma. We believe that bioengineered organoids supported by smart bio-printed tubules and microfluidic organ-on-a-chip systems offer efficient, precise, personalized platforms for autologous pluripotent stem cell sources to undergo the spermatogenetic cycle, presenting a promising tool for infertile male patients with complete testicular aplasia.

**Graphical Abstract:**

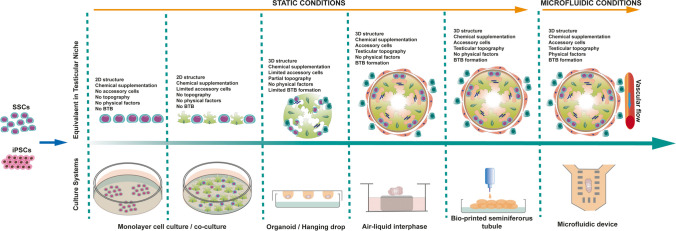

## Introduction

Worldwide twelve to fifteen percent of couples are diagnosed with infertility, and approximately half of these cases are due to azoospermia in males. Azoospermia is characterized by the absence of sperm in the ejaculate and its incidence rate is 10–15% in infertile males [1]. Congenital anomalies and later acquisitions can lead to partial or permanent infertility in males. In case of low sperm or elongated/round spermatid production in testis, assisted reproductive techniques (ART), such as intracytoplasmic sperm injection (ICSI) and round spermatid injection (ROSI), offer a well-defined alternative to these patients. However, there is no effective treatment option for patients with germ cell aplasia or spermatogenic arrest in the earlier development than the round spermatid stage [2].

In limited preclinical studies, human induced pluripotent stem cells (hiPSCs) exhibited a significant treatment potential for male germ cell aplasia [3–10]. iPSCs reprogrammed from dermal fibroblasts [3, 11–15], cord blood, and keratinocytes [16] of healthy, non-obstructive azoospermic (NOA) men with an azoospermia factor 1 (AZF) deletion showed the capacity to differentiate into spermatogonial stem cells (SSCs), spermatogonial stem/progenitor cells, spermatocytes and spermatids in monolayer and air–liquid interface (ALI) culture setups. Those studies reported an efficiency of human SSCs (hSSCs) generation from iPSCs in culture systems with a wide variation ranging from 20 to 80%. The success rate of obtaining haploid germ cells is very limited, varying from 0 to 14%, and can therefore not be translated to ART applications.

These insufficient outputs underline the need for the development of 3D dynamic ex vivo setups that model the in vivo physical and chemical microenvironment of SSCs in testicular seminiferous tubules [17, 18]. The expected outcome is to enable progenitor germ cells to reach a number for spermatogenetic initiation sufficient for production of mature and functional sperm at satisfactory yields. Therefore, recent studies have tested in vitro spermatogenesis and testicular maturation in human samples using 2D and 3D culture systems, including monolayer, ALI, soft agar, testicular organoid, and microfluidic platforms (Table 1). The success of the present experimental designs mainly relies on niche-based concepts but faces long-term failure in the absence of a dynamic platform simulating the microvascular 3D physiology of the seminiferous tubules. Therefore, the development of a novel biomedical transdisciplinary approach that recapitulates the embryologic development of male germ stem/progenitor cells within the parenchymal and stromal testicular compartments, providing an effective cell-to-cell and cell-to-ECM crosstalk under appropriate physical conditions, is crucial for clinical translation. This article aims to provide a road map for researchers and health professionals working on male infertility to find a niche-based solution for the challenging progress of ex vivo spermatogenesis based on our group’s work history and other recent trials.

**Table 1:** Current studies on human in vitrospermatogenesis platforms are summarized

Culture Platform	Experimental Setup	Obtained Cells/*Yield*	Ref
Organ culture-air–liquid interface	21-day culture of testicular strips taken before treatment from prepubertal (7, 9, and 12 years old) male cancer patients	• PIWIL4+ , VASA+ SSCs • BOULE+ spermatocytes • SMA+ , LAMA1+ peritubular cells • SOX9+ , CLDN11+ Sertoli cells • STAR+ , CYP17A1+ , INSL3+ Leydig cells *50- 70% decrease in hSSCs*	[54]
Culture in 3D soft agar	Culture of hSSCs from postmortem testicles of healthy post-pubertal (15, 21, 26 years old) donors for 74 days in a monolayer, 3D Transplantation into busulfan-treated mouse testis	• PLZF+ hSSCs • SCP3+ elongated spermatids *Haploid cell rate was 0.61% in monolayer, 2.91% in 3D culture (increase threefold compared to monolayer)*	[66]
Culture of testicular organoid and seminiferous tubule samples created with bio-printer on U-plate	Organoid and bio-printer-based culture of the testicular cell suspension (Sertoli / Leydig / endothelial cells) obtained from a post-pubertal 31-year-old SCO patient with partial germ cell aplasia for 12 days	• ID4+ , FGFR3 + , c-Kit + , STRA8 + hSSCs • SYCP3+ spermatocytes • TP1+ , PRM2 + spermatids • SOX9+ Sertoli cells • INSL3+ Leydig cells • ACTA2+ peritubular myoid cells *In the bio-printed seminiferous tubule, a fourfold increase in the number of ID4*+ *hSSC, a 2-fold increase in the number of SCP3*+ *spermatocytes, and a decrease in the number of PRM2*+ *spermatids compared to organoid*	[63]
Organ culture air–liquid interface	Culture of frozen immature testicle strip from a prepubertal (6 months—1.4 years old) bilateral cryptorchidism patient with RA-supplemented medium for 60 days	• MAGE-A+ , GAGE+ , VASA+ spermatogonia • BOLL+ spermatocytes • SOX9+ , AMH+ , AR+ Sertoli cells • STAR+ Leydig cells • ACTA+ peritubular myoid cells *50–70% decrease in spermatogonia, presence of BOLL*+ *spermatocytes*	[55]
Monolayer direct co-culture on gelatin coated plates	7-day culture of allogeneic fetal testis-derived Sertoli cells and hSSCs with β-estradiol supplemented medium	• c-Kit+ and SSEA4+ hSSCs *Yield increased 2–4 fold*	[49]
Organ culture air- liquid interface	Isolation and culture of haploid cells from strips taken from the gonads of 45 curetted fetuses (between 12–19 weeks) for 50 days in the ALI system ROSI with resulting round spermatids Embryo development	• DDX4+ germ cells • PLZF+ hSSCs • SYCP3+ spermatocytes • PRM1+ spermatids • SOX9+ Sertoli cells • CYP17A1+ Leydig cells *Haploid cells developed in 14 samples with a yield of 0.069-* *9.83%*	[53]
3D soft agar culture system	Co-culture of testicular cells isolated from healthy postmortem post-pubertal (15, 21, and 26 years old) male testicles with Sertoli cells in a monolayer Culture of obtained hSSCs in agarose and laminin in the 3D soft culture system for 3 weeks	• PLZF+ and c-Kit+ hSSCs *Increased SSC yield, further spermatogenic differentiation* *not evaluated*	[67]
3D Allogeneic PRP-based tissue scaffold	Co-culture of testicular cells isolated from healthy postmortem post-pubertal (15, 21, and 26 years old) male testicles with Sertoli cells in monolayer Culture of hSSCs seeded on the PRP tissue scaffold and cultured for 2 weeks	• GFRa1+ and c-Kit+ hSSCs *The yield of hSSCs increased 2–4 times compared to monolayer culture; further differentiation is not evaluated*	[68]
Testicular organoid	5-day culture of prepubertal (5-year-old) human testicular cell-derived organoids Comparison with pig, mouse, macaque organoids	• GATA4+ Sertoli cells • aSMA+ peritubular myoid cells *No evaluation of germ cells*	[60]
3D soft agar culture system	5-week culture of testicular cells from fresh/frozen-thawed testicles of 9 pre-pubertal boys (6–14-year-old)	• MAGE4+ and PCNA+ hSSCs *hSSCs yield increased by 50%; further differentiation is not evaluated*	[56]
Air–liquid interface organ culture	Isolation of GPR125 + hSSCs and Sertoli cells from a testicular biopsy taken from 80 post-pubertal OA patients between the ages of 13–47 Cultured in Matrigel for 20 days	• GPR125+ , GFRA1+ , UCHL1+ , PLZF+ , MAGEA4+ hSSCs • SYCP3+ , ACR+ spermatids *Haploid cell yield is between 0.8–17.9%*	[57]
Organ culture with multi-well plate insert	Pre-treatment of testicular strips from 5 prepubertal cancer patients (2–12-year-old) by freeze/thaw Culture for 139 days	• MAGE4+ hSSCs • SOX9+ , AMH+ Sertoli cells • SYCP3+ spermatocytes • BOLL+ round spermatids • ACR+ elongated spermatids *The number of hSSCs decreased by 50–80%; spermatids preserved throughout the culture*	[69]
3D soft agar culture system and 2D gelatin coated system	Pre-treatment of testicular strips from 4 prepubertal cancer patients (7–14-year-old) Culture for 70 days	• DAZL+ , PLZF+ , FGFR3+ , SALL4+ hSSCs • SYCP3+ , ACR+ haploid germ cells *SSCs decreased by 0.5–4 times, and haploid germ cells decreased by more than 5 times*	[70]
3D soft agar culture system (SACS) and 2D gelatin-coated system	Collection of hSSCs obtained from testicular biopsies taken from 14 post-pubertal (21- 41 years old) male patients with NOA Culture in the 3D soft agar culture system for 2 weeks	• STRA8+ hSSCs • SCP3+ , Acrosin+ haploid germ cells *In 3D condition, hSSCs increase 2 times and haploid germ cells increase 2–3 times compared to 2D*	[58]
Testicular clusters	Culture of testicular cells obtained from 16 post-pubertal (between 28 and 58 years of age) patients’ (impaired spermatogenesis) TESE samples for 3 weeks	• SOX9+ , SMA+ , PRM1+ clustered mixed cell population *Quantitative data is not reported*	[71]
Testicular organoid	23-day culture of testicular tissue isolated from post-pubertal (56–71 years old) healthy donors	• PLZF+ , UCHLI+ , THY1+ hSSCs • DAZL+ , PRM1+ , ACR + post-meiotic germ cells • GATA4+ , CLU+ , SOX9+ Sertoli cells • STAR+ , TSPO+ , CYP11A1+ Leydig cells • CD34+ , ACTA2 + peritubular cells *Yields of hSSCs are 52%, post-meiotic germ cells are %0.2*	[61]
Testicular organoid	Culture of testicular cells obtained from 6 donors (performing all spermatogenesis) after bilateral orchiectomy	• DDX4+ , FGFR3+ , UTF1+ , UCHL1+ hSSCs • SOX9+ , ZO1+ Sertoli cells • STAR+ Leydig cells • ACTA2+ , COL1+ myoid cells *Quantitative data is not reported*	[72]
Organ culture—culture with multi-well plate insert	Isolation of immature testicular tissue from testicular biopsy taken from boys (aged 2, 11, and 12 years) before prepubertal cancer treatment Culture in the insert system for 139 days	• MAGE4+ hSSCs *Increase in hSSCs, further differentiation not evaluated*	[73]

*hiPSCs: Human induced pluripotent stem cells, hESCs: Human embryonic stem cells, hPGCs: Human primordial germ cells, hSSCs: Human spermatogonial stem cells, hSPCs: Human spermatogonial progenitor cells*

## Method

A detailed literature review was carried out in PubMed using the keywords “in vitro spermatogenesis”, “testis organ culture”, “testis organoid”, “induced pluripotent stem cell/iPSC”, “Very small embryonic- like cells”, “male infertility”, and “germ cell aplasia”. The references section of this review includes the articles on in vitro human male germ cell differentiation published in English. The output of our group’s focused experience over ten years on stem cell niche, male infertility, and organ-on-a-chip models is cited and has been used to draw a guiding roadmap for future niche-based therapeutic concepts to male infertility research.

## Male Factor Infertility and Germ Cell Aplasia

Infertility is the failure to achieve a successful pregnancy, as mentioned by the American Society for Reproductive Medicine (ASRM) in 2023 [19]. It is recommended that couples be followed up for infertility when pregnancy does not occur after 12 months of unprotected sexual intercourse if the female partner is under 35 years of age or six months of unprotected sexual intercourse if the female partner is 35 years of age or older. Infertility occurs in nearly 12–15% of couples and approximately 50% of these cases are due to male factors [20, 21]. Male factor infertility (MFI) is classified as congenital and acquired according to the point of origin in patients. Although MFI can be congenital with anomalies, such as bilateral absence of *vas deferens*, Sertoli Cell-Only (SCO) syndrome, cryptorchidism, Y chromosome microdeletion and Klinefelter syndrome, it can also be acquired at various stages of life due to environmental factors, such as testicular torsion, cancer treatment, trauma, multiple diseases or cytotoxic treatments [22, 23]. In case of bilateral absence or obstruction of the *vas deferens*, spermatogenesis continues normally, and functional sperms are produced in the testis; however, due to the lack of connection between the epididymis and urethra, the semen is devoid of sperm. As a routine practice of ART, sperm is collected from these patients by interventions, such as testicular sperm extraction (TESE) or micro-TESE followed by ICSI for pregnancy [24]. Similarly, cancer treatments, such as chemotherapy and radiotherapy, have gonadotoxic effects and can stop spermatogenesis in the testicle irreversibly. For male patients diagnosed with cancer in late adolescence or adulthood, mature sperm samples can be isolated before cytotoxic treatment within the scope of ART and pregnancy may be achieved through ICSI [25, 26]. There are several options in the clinic for treatment of infertility problems, where healthy spermatogenesis proceeds. However, these solutions are limited to patients having mature and functional sperm production.

**Fig. 1:**
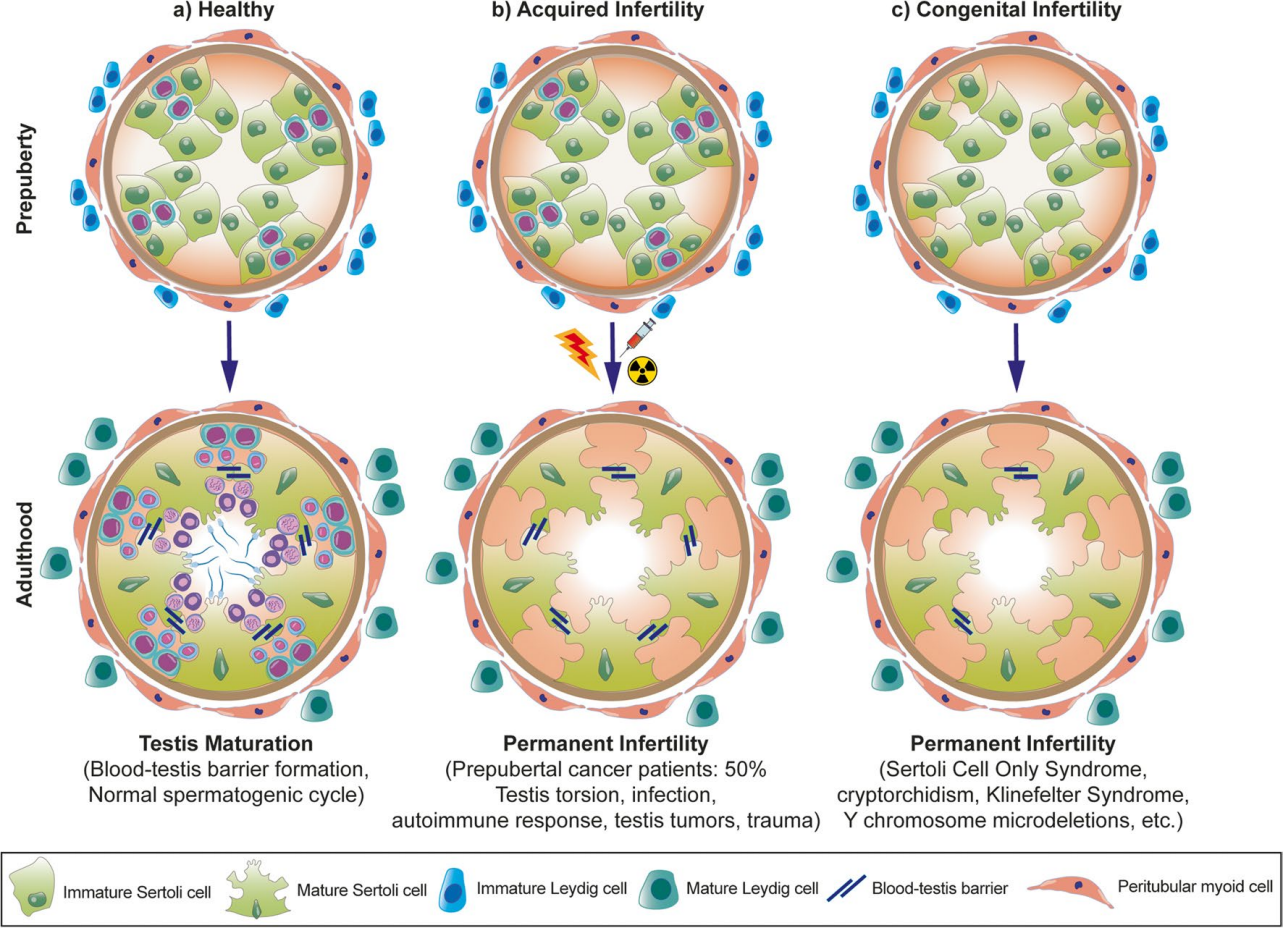
The illustration depicts the testicular seminiferous tubule microenvironment, referred as the “SSC niche” in (**a**) healthy conditions, (**b**) acquired and (**c**) congenital anomalies in prepuberty and adults

Congenital anomalies, such as SCO syndrome, cryptorchidism, Klinefelter syndrome or Y chromosome-microdeletion [27] and acquired causes, such as testicular trauma, childhood cancer treatment, testis torsion, autoimmune response and viral infections [28] cause arrests in the early stages of spermatogenesis before spermatid or complete germ cell loss in testes (Fig. 1).

The patients face permanent infertility since no improved treatment procedure can provide pregnancy. Since the cellular and physical integrity of the germ cell niche is impaired in male patients with germ cell aplasia, regardless of etiological factors, they cannot conceive a biological child. Because the spermatogenic cycle cannot be initiated, these males are not able to produce mature sperm or even late-stage spermatids. Recently, in vitro spermatogenesis is targeted with niche simulation using germ stem cell, organ, organoid or bioprinting-based culture platforms for patients with a damaged/impaired testicular milieu.

## The Spermatogonial Stem Cell Niche for in vitro Spermatogenesis

The spermatogonial stem cell lineage localizes and acts within a unique microenvironment, resembling that of somatic stem cells [29, 30]. The testicular microenvironment of the SSCs comprises several accessory cellular and extracellular matrix elements which interact within a physical and chemical milieu. The overall zone that encourages the self-renewal of male germ stem cells to preserve spermatogenic cell pool at adequate size, is referred to as “niche”. Since spermatogonial stem cells constitute only 22% of the niche in humans [31] and 0.01- 0.02% in mice [32], the accessory elements comprise the major fraction. The spermatogonial stem cell niche (Fig. 1. a) is placed at the seminiferous tubules lined by a germinal epithelium that forms the parenchyma and takes a part of the stroma between the tubules in the testes [33]. The spermatogenic stem cells remain undifferentiated due to lack of androgenic endocrine induction but maintain the germ cell pool within the human testicular cords until puberty. The somatic Sertoli cells (SCs) assist germ cell renewal and provide the blood-testis barrier during childhood.

By the onset of the puberty, spermatogenic cell series that start from the basement membrane (BM) present a stratified epithelial-like tissue when they undergo differentiation with the support of somatic SCs. The interstitial or stromal testicular compartment comprises Leydig cells (LCs), peritubular myoid cells (PMCs), macrophages and blood vessels. Sertoli cells are highly polarized and cytoplasmic projections of SCs laterally extend to the lumen of seminiferous tubules while their basal region is located on the BM [33, 34]. The somatic cells support self-renewal and survival of SSCs in many ways. Sertoli cells form a blood-testis barrier and produce glial cell line-derived neurotrophic factor (GDNF) and fibroblast growth factor 2 (FGF2) to provide structural and homeostatic support to the SSC pool. Leydig cells are androgenic cells after puberty. They are activated by luteinizing hormone (LH) and secrete testosterone, which is responsible for stimulation of spermatogenic lineage cells to the terminal stage. Both LCs and PMCs produce colony-stimulating factor 1 (CSF1) to conserve SSC self-renewal [29, 34, 35]. Experimental ex vivo testicular niche setups require the presence of both parenchymal and stromal compartments that physiologically act in harmony with neural, paracrine, endocrine, and vascular functions to obtain fertile spermatozoa.

**Fig. 2:**
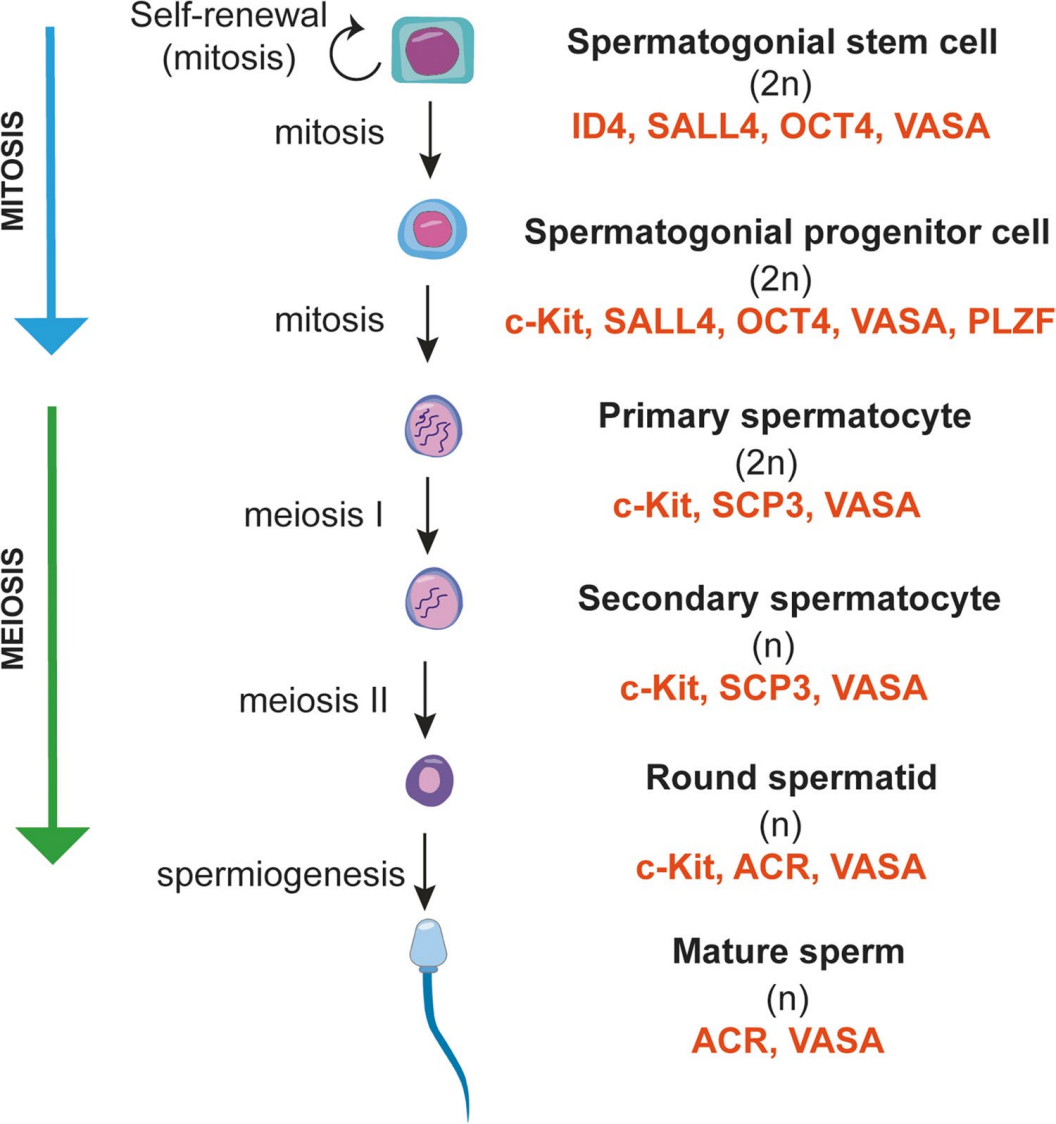
The illustration shows spermatogenesis with characteristic markers of spermatogenic lineage cells

Spermatogenesis comprises consecutively spermatogonial, spermatocyte and spermatid phases (Fig. 2). One cycle of human spermatogenesis lasts about 64 days [36] and starts with the spermatogonial phase due to SSC mitosis into two daughter cells [37]. One of the daughter cells continues mitosis to preserve self-renewal of SSCs and the other one begins differentiation [37]. The new cycle of spermatogenesis can start when the previous cycle is completed [36]. The daughter cells of the SSCs are classified into three types based on their nuclear histochemical staining intensity as type A_dark_, type A_pale_ and type B spermatogonia. Type A_dark_ spermatogonia are responsible for the SSC pool and undergo mitotic division. Type A_pale_ spermatogonia differentiate to form type B spermatogonia [38–40]. Type A_dark_ and B spermatogonia are connected to each other with cytoplasmic bridges and these connections continue until the spermatid phase. In the spermatocyte phase firstly, type B spermatogonia mitotically divide to generate primary spermatocytes with a normal chromosome number. Whereas spermatogenesis occurs in the basal region, the remainder occurs in the lumen of the seminiferous tubules. Then, primary spermatocytes (2n) undergo their first meiotic division in order to obtain haploid DNA (n) while forming secondary spermatocytes (Fig. 2). After the second meiotic division, haploid spermatids are produced [38, 41]. Finally, spermatids undergo morphological remodeling to form mature sperms in the spermatid phase (Fig. 2). In this phase, spermatids undergo Golgi, cap, acrosome, and maturation phases, after which spermatogenesis is completed. The mature sperms are released from the basal region of SCs to seminiferous tubules with the help of PMCs [38].

In the last decade, the presence of very small embryonic-like cells (VSELs) has been reported in both the pre- and post-pubertal human testicular microenvironment [42–44]. The spherical shaped VSELs are overlooked in many studies due to their very small-size (range of 2- 6 µm diameter) [45]. However several groups define the human VSELs located along the basal lamina as similar to the SSCs, but more primitive due to the presence of the pluripotency markers, such as OCT4, NANOG, SOX2, LIN-/CD45-/CD133 + , SSEA4 + , SSEA1 + , and a high nucleocytoplasmic ratio at the ultrastructural level [44, 46]. Human testicular primordial germ cell like VSELs are physiologically quiescent, however they can rapidly divide and give rise to SSCs when exposed to stress-inducing conditions. This may present a potential tool for the SSC enrichment tools [45, 47]. Ex vivo spermatogenesis frameworks require the completion of the whole orchestrated process of germ stem cell differentiation and the generation of a sufficient number of functional sperms in order to achieve efficient fertility incomes in the clinic.

## The Evolution of Testicular Culture Platforms

To create new treatment options for congenital and acquired male infertility, there is a need for systems that model the 3D hSSC niche with active neural inputs, paracrine/endocrine secretome and a continuous vascular flow of the testis. When spermatogenesis cannot start during puberty because of damaged SSCs and other cellular and extracellular niche components due to chemotherapy and/or radiotherapy for prepubertal cancers, clinically permanent infertility may be observed in half of these patients. In countries with fertility preservation programs, testicular biopsies are usually taken from patients before gonadotoxic therapy, depending on their parents' approval. The aim is to protect the existing SSCs via cryopreservation. However, in a small population of patients with a damaged testicular microenvironment due to cancer therapy, fertility cannot be achieved from the cryopreserved SSCs [25, 26, 48]. Therefore, in vitro expansion and differentiation of the isolated SSC pool is crucial for restoration of fertility. However, because the self-renewal and spermatogenic differentiation process is highly niche dependent for SSCs, this may hinder the success of ex vivo spermatogenesis.

Monolayer stem cell cultures in culture plates coated with Matrigel/Fibronectin or a feeder layer (i.e. mouse embryonic fibroblasts) have been shown to provide short-term maintenance for rodent [49] and human [50] SPC lineages. A β-estradiol-supported monolayer germ stem cell-Sertoli cell co-culture system increased hSSC number by physical contact and intercellular crosstalk with SCs and extrinsic Leydig cell-based chemical factor supplementation, when compared to monoculture of SSCs until day 7 [49]. Addition of epididymal white adipose tissue (EWAT)-derived leptin showed limited performance by increasing colony formation and real-time proliferation rate when used in to expand newborn mouse SPCs that are cultured on mouse embryonic fibroblasts until day 7 [51]. The co-culture of allogeneic adult EWAT with newborn mouse prepubertal testicular strips on an ALI system provided a longer survival and enrichment of SPCs until day 14. EWAT secretome also supported the differentiation of SSCs up till the round spermatid stage in a diffusion permeable static organ culture setup. The addition of allogeneic mouse bone marrow mesenchymal stem cells (BMSCs) to the ALI co-culture system enhanced the success of IVS in terms of SSC proliferation and SCP3 and acrosin-positive haploid germ cell formation, which increased up to 28 days. Considering the ALI platform’s ability to simulate SSC niche, allogeneic BMSCs having a similar embryonic origin are able to replace and support SCs that are essential for the engraftment and differentiation of spermatogenic cells. Thus, they preserve the integrity of tight junctions and exert a paracrine support by GDNF, FGF, stem cell factor (SCF), TPO and vascular endothelial growth factor (VEGF) [52]. Human fetal gonad strips obtained from 12 to 19-week-old abortus material were maintained in this ALI platform for 50 days and allowed the differentiation of haploid cells, but at a limited efficiency, ranging between 0.07 to 9.83%. When ROSI was performed with these haploid cells, resulting embryos were shown to develop until the blastocyst stage [53]. Air–liquid interphase cultures provide an inadequate yield of haploid germ cells or complete absence of haploid germ cell production after 5 to 139 days of culture of human testicular biopsies from prepubertal cancer patients [54–56]. When GPR125 positive SSCs and SCs isolated from testicular biopsies from 80 post-pubertal obstructive azoospermia patients with normal sperm production were embedded into Matrigel and cultured via the ALI platform for 20 days, the system yielded haploid cells with 0.8 to 17.9% efficiency [57]. Spermatogonial stem cells obtained from testicular biopsies taken from 14 post-pubertal (21- 41 years old) male patients with non‐obstructive azoospermia (NOA) were cultured in agarose for two weeks in the 3D soft agar culture system. 3D soft agar conditions increased hSSCs two times and haploid germ cells increased 2–3 times compared to 2D monolayer culture [58], clearly demonstrating a need for novel organ culture designs.

In the last decade, organoid, bioprinter-based seminiferous tubule and testicular cell aggregate culture systems have replaced monolayers for simulation of the healthy spermatogenic microenvironment outside the body and to obtain mature and functional sperm from these patients (Table 1). Organoids can be generated through culture of pluripotent or primary niche cells. The aggregation of those cells resulting in adhesion, self-organization and differentiation into 3D cell masses might represent the corresponding organ’s functional morphology [59]. A 5-day culture of organoids obtained from prepubertal pig, macaque, and mouse testicular samples under static conditions resulted in adhesive connections of SCs and PMCs but was unable to sustain the germ cell pool [60] (Table 1). Post-pubertal healthy donor testis organoids yielded hSSCs with 52% efficiency after 23 days of culture, while the yield of haploid cells was limited to 0.2% [61]. Bioprinting is the fabrication of complex biological constructs regarding cellular/extracellular content and tissue topography, including tube-shaped architecture of seminiferous tubules [62]. The cell suspension of SSC/Sertoli/Leydig/endothelial cells obtained from a testicular biopsy of an adult SCO patient with partial germ cell aplasia was seeded into a 3D bio-printed seminiferous tubule or aggregated as an organoid, followed by culture for 12 days. The study reported a fourfold increase in the number of ID4 positive hSSCs, a twofold increase in SCP3 positive spermatocytes and a decrease in the number of PRM2 positive spermatids in the bio-printed seminiferous tubule compared to the organoid [63]. Taken together, 3D smart bioengineered materials present promising tools, when combined with amassed cell-sourced organoids mimicking embryologic nest topography.

In conclusion, ex vivo culture conditions have evolved from basic monolayer SSC cultures containing only the stem cells and an artificial chemical microenvironment generated by supplementation, devoting of physical contact, and crosstalk with neighbor cells within the SSC niche to static organ culture systems [51]. These systems comprise ALI and hanging drop setups and the bioengineering product-based scaffolds. Briefly ALI permits a homogenous diffusion through a biphasic compartment, while the hanging drop system avoids the damage by immersion of the organ strip in growth factor containing media [10, 41, 64]. The bioengineering products such as natural and synthetic (alginate) polymers that make 3D immersion setups replace the ECM of the stromal compartment of the niche model and mimic the testicular microenvironment ex vivo. These static setups caring seminiferous tubule strips are highly similar to the SSC niche in terms of collecting spermatogenic cells, accessory cells (Sertoli cell, Leydig cell, PMC, etc.), secretome based on physical contact and intercellular crosstalk (chemical factors). However, they lack several crucial physical elements such as vascular flow, shear stress, O_2_ gradient, and equal distribution of nutrients to growing seminiferous tubules. These shortcomings necessitated the development of real organoids and organ-on-a-chip systems that involve differentiation of pluri/multipotent stem cells within a dynamic 3D milieu. Our group recently designed and generated a factitious microfluidic compartment mimicking not only cellular and chemical factors, but also the physical elements in the SSC niche with a pumpless flow in microchannels, polydimethylsiloxane (PDMS)-controlled O_2_ gradient and diffusion-based nutrient distribution to the seminiferous tubules through an array of rectangular micropillars and bilateral medium perfusion around an organ chamber [17, 65]. In the microfluidic device designed with a holistic niche concept and supported by allogeneic BMSCs secretome, the duration of the culture period was increased to 42 days, and thus the artificial niche environment could be maintained throughout a complete cycle of mouse spermatogenesis, which lasts for 34 days under in vivo conditions. The actual success rate achieved from current culture systems may encourage infertility professionals to treat patients who already have a healthy SSC niche and/or healthy SSC population, but patients with complete germ cell aplasia still require a novel germ cell source. For this reason, a few groups, including ours, are currently conducting studies on the differentiation of human ESCs and iPSCs from autologous sources into SSC and haploid germ cells.

## Pluripotent Stem Cell-Based Culture Technology as an Opportunity to Restore Germ Cell Aplasia

**Fig. 3:**
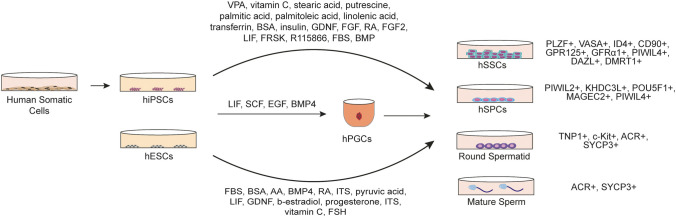
The schematic illustration of the current in vitro male germ cell differentiation protocols from iPSCs. hiPSCs: Human induced pluripotent stem cells, hESCs: Human embryonic stem cells, hPGCs: Human primordial germ cells, hSSCs: Human spermatogonial stem cells, hSPCs: Human spermatogonial progenitor cells, VPA: Valproic acid, BSA: Bovine serum albumin, GDNF: Glial cell line-derived neurotrophic factor, FGF: Fibroblast growth factor, RA: Retinoic acid, LIF: Leukemia inhibitory factor, FRSK: Forskolin, FBS: Fetal bovine serum, BMP: Bone morphogenetic protein, SCF: Stem cell factor, EGF: Epidermal growth factor, AA: Activin A, ITS: Insulin-Transferrin-Selenium, FSH: Follicle stimulating hormone

Currently, methods for in vitro generation of male germ cells have generally been based on pluripotent stem cells (PSCs), due to their plasticity (Fig. 3). Both ESCs and iPSCs are classified as pluripotent stem cells. ESCs are collected from the inner cell mass of the blastocyst stage and have the full potential to differentiate into cells from ectoderm, mesoderm and endodermal germ layers [74]. However, due to ethical and allogenic restrictions their use is limited, even though their differentiation potential makes ESCs a good candidate for in vitro male germ cell differentiation [75–77]. iPSCs are reprogrammed from somatic cells and gain a pluripotent profile, similar to ESCs [78]. There are two ways to conduct this process: chemically induced supplements or vectors, which can either be non-integrating or integrating. The efficiency of integrating viral vectors is higher when compared to induction via chemicals and the use of non-integrating vectors. However, viral genomic integration into the host cell genome may cause unpredictable and permanent mutagenesis [79]. Since VSELs are reported to be adult testicular primitive primordial germ cells or putative PSCs [42, 45, 47, 80] they may therefore be worth further assessment, as an alternative source for the infertility studies [47]. In this context, recently non-integrating vectors presenting higher safety profiles have come forward in iPSC manufacturing technology and could be used in autogenic or allogeneic stem cell-based therapies, including male fertility approaches [81, 82].

Both human ESCs (hESCs) and hiPSCs have been differentiated to male germ cells by various in vitro methods, but with a very low yield (Fig. 3, Table 2) [3, 5, 11–13, 15, 83]. Human iPSCs are induced with secretomes of Sertoli cells (bFGF, GDNF), EWAT (palmitic acid, palmitoleic acid, linoleic acid, valproic acid), putrescine, insulin, transferrin, sodium selenite, and vitamin C to differentiate into SSCs [3, 11, 84, 85]. Human iPSCs have been stimulated with GDNF, retinoic acid (RA), bone morphogenetic protein (BMP) 4, BMP8b, leukemia inhibitory factor (LIF), forskolin (FRSK) and R115866 to differentiate to haploid germ cells [5, 12]. In a recent study, commercial hESC lines and NOA-patient primary dermal fibroblast-derived iPSCs are evaluated for their differentiation capacity into the SSCs using valproic acid (VPA) and vitamin C. Human PSCs are induced by knockout serum replacement (KSR), GlutaMAX™, Insulin-Transferrin-Selenium (ITS), lipid mix, GDNF, bFGF, VPA and vitamin C for 12 days. Application of VPA and vitamin C together increases PLZF + , GPR125 + , GFRa1 + , ID4 + SSCs and SYCP3 + , Acrosin + , TNP1 + haploid germ cells [11]. These studies suggest iPSCs may be a promising source of SSC and haploid germ cells, however, the haploid germ cell yield from iPSCs is currently limited to a range of 0–5% in monolayer, static culture systems.

**Table 2:** Differentiation studies of hESCs and hiPSCs to male germ cells in vitro platforms are summarized

Cell Type	Platform Type	Study Design	Results/*Yield*	Ref
hESCs line and iPSCs derived from dermal fibroblasts of NOA patient	Monolayer	Differentiation of SSCs from iPSCs with VPA and vitamin C supplementation	• *PLZF*+ *, VASA*+ *, ID4*+ *, GFRa1*+ hSSCs • *SYCP3*+ *, Acrosin*+ *, TNP1*+ haploid cells *Low yield with 2–5% haploid germ cells*	[11]
iPSCs derived from somatic cells	3D	Differentiation of PGCs from iPSCs then aggregating with SSEA + foetal mouse testicular cells	• *DAZL*+ *, DND1*+ *, PIWIL2*+ prospermatogonia • *KHDC3L*+ *, POU5F1*+ M- pro-spermatogonia • *MAGEC2*+ *, PIWIL4*+ T1- pro-spermatogonia *Yield is not reported*	[83]
hESCs line and iPSCs derived from foreskin fibroblasts of healthy donor	Monolayer	Differentiation of SSCs from hiPSCs with stearic acid, putrescine, palmitic acid, palmitoleic acid, linolenic acid, transferrin, BSA, insulin, GDNF, FGF supplemented medium for 6 days at 37^o^C then culture for 18 days at 34^o^C	• *PLZF*+ *, CD90*+ *, GPR125*+ *, VASA*+ *and GFRα1*+ h*SSCs* *Yield is not reported*	[85]
iPSCs derived from dermal fibroblast of healthy donor	Monolayer	Differentiation of haploid germ cells from iPSCs with xenofree culture condition	• *PLZF*+ *, GPR125*+ *, CD90*+ *, PIWIL4*+ *,* *DAZL*+ *, DMRT1*+ *, ID4*+ *, GFRa1*+ hSSCs • *TNP1*+ *, SYCP3*+ *, Acrosin*+ haploid germ cells *Low yield with 5% haploid germ* cells	[12]
iPSCs derived from dermal fibroblasts of azoospermia patient	Monolayer	Reprogramming of fibroblasts with OSKM factors and VASA transcription factors	• *VASA*+ *, GFRa1*+ *, DAZL*+ *, UTF1*+ hSSCs *Yield is not reported*	[15]
hESCs line	3D	Induction of ESCs with FBS, BSA, AA supplemented medium to obtain EB for 8 days Culture of EBs with BMP4, RA, ITS, pyruvic acid, LIF, GDNF, b-estradiol, progesterone, and isolation of GFRa1+ cells Embedding the GFRa1+ cells in alginate and incubation with ITS, vitamin C, FSH, RA, retinol, testosterone for 6 weeks	• *cKit*+ h*SSCs* • *TH2B*+ *spermatocytes* • *TP1*+ *spermatids* *Yield is not reported*	[84]
iPSCs derived from dermal fibroblast of healthy donor	Monolayer	Differentiation of hSSCs from iPSCs with mouse SSCs induction medium	• *VASA*+ *, DAZL*+ *, PLZF*+ *, UTF1*+ hSSCs • *SYCP3*+ spermatocytes • *TNP1*+ *, Acrosin*+ spermatids *Yield of hSSCs and haploid germ cells are 79% and 3.9%, respectively*	[3]
iPSCs derived from cord blood and keratinocytes	Monolayer	Culture of iPSCs with FGF2 and RA-supplemented PSC medium for three weeks Isolation and culture of CD49f+ , CD9+ , CD90-, SSEA4- cells with FGF2, LIF, FRSK and R115866 supplemented medium for four weeks	• VASA+ germ cells • *SYCP3*+ *, Acrosin*+*, γH2AX*+ *haploid germ* *cells* *Yield is 0- 2.33% and 0- 1.98% in cord blood and keratinocytes groups, respectively*	[5]
iPSCs derived from dermal fibroblasts	Monolayer	Culture of iPSCs with FBS and BMP-supplemented medium Transduction with VASA: GFP reporter	• *Acrosin*+ spermatids *Yield is 1.6- 14%*	[13]

*hiPSCs: Human induced pluripotent stem cells, hESCs: Human embryonic stem cells, hPGCs: Human primordial germ cells, hSSCs: Human spermatogonial stem cells, hSPCs: Human spermatogonial progenitor cells, VPA: Valproic acid, BSA: Bovine serum albumin, GDNF: Glial cell line-derived neurotrophic factor, FGF: Fibroblast growth factor, RA: Retinoic acid, LIF: Leukemia inhibitory factor, FRSK: Forskolin, FBS: Fetal bovine serum, BMP: Bone morphogenetic protein, SCF: Stem cell factor, EGF: Epidermal growth factor, AA: Activin A,*

*ITS: Insulin-Transferrin-Selenium, FSH: Follicle stimulating hormone*

Taken together, monolayer culture has been widely used in studies targeting in vitro spermatogenesis from iPSCs, but only two studies used cell aggregates and EB-based 3D platforms [83, 84]. We foresee that the experience we have gained from our niche concept-based work history will serve as a crucial milestone to increase the yield of SSCs and haploid germ cells from iPSCs.

## Conclusion

Repeated clinical failure has directed the current strategy to ensure functional testicular maturation, SSC proliferation and spermatogenesis by in vitro culture of prepubertal testicular samples containing hSSCs and immature Sertoli, Leydig cells and myoid cells. From another perspective, spermatogenesis might be arrested before germ cells become haploid and gain fertilization potential, due to impairment of niche components and blood supply disruption such as in cases of testicular torsion, trauma, varicocele and infection in adulthood [22]. The overall preclinical experience reveals the capacity of 3D culture platforms to support in vitro spermatogenesis from 5 to 139 days. The 3D organ, organoid, and bio-printed testicular static cultures give limited yields and differentiation capacities with insufficient numbers of post-meiotic germ cells. The evolution from monolayer to 3D in vitro spermatogenesis platforms improved the yields to some extent, especially when microfluidic flow enforced allogeneic BMSC secretome was used within a 3D dynamic tubular topography.

However, generation of a continuous supply with a high yield of fertile spermatozoon requires reprogramming of pluripotent stem cells. Recently, pluripotent stem cell-based culture technology has supported the possibilities to restore somatic and also germ cell pools. Although autologous iPSCs may present a promising tool for SSCs this method currently only gives a very limited yield of haploid germ cell production in the range of 0–5%, underlining that the present culture systems are insufficient for clinical translation of treatment for germ cell aplasia. We propose that stem cell-assisted microfluidic-based platforms could be used to overcome this boundary in the near future, provided that smart bioengineering meets the micro-physiological needs. Besides functionality, the genetic stability of ex vivo differentiated haploid cells and the embryos needs to be validated before transferring these systems to the clinic. Our evolutionary niche-based road map could guide future translation of stem cell therapeutics into the male infertility clinic and may be used as a precise personalized tool by exploring the possibilities of 3D organ-on-a-chip platforms.

## Data Availability

Not applicable.

## Abbreviations

AA: Activin A
ALI: Air-Liquid Interface
ART: Assisted Reproductive Techniques
ASRM: American Society for Reproductive Medicine
BM: Basal Membrane
BMP: Bone Morphogenetic Protein
CSF1: Colony-Stimulating Factor 1
EGF: Epidermal Growth Factor
ESCs: Embryonic Stem Cells
FBS: Fetal Bovine Serum
FGF: Fibroblast Growth Factor
FRSK: Forskolin
FSH: Follicle Stimulating Hormone
GDNF: Glial Cell Line-Derived Neurotrophic Factor
ICSI: Intracytoplasmic Sperm Injection
iPSCs: Induced Pluripotent Stem Cells
ITS: Insulin-Transferrin-Selenium
KSR: Knockout Serum Replacement
LCs: Leydig Cells
LIF: Leukemia Inhibitory Factor
MFI: Male Factor Infertility
NOA: Non-Obstructive Azoospermia
PGCs: Primordial Germ Cells
PMCs: Peritubular Myoid Cells
PSCs: Pluripotent Stem Cells
RA: Retinoic Acid
ROSI: Round Spermatid Injection
SCs: Sertoli Cells
SCF: Stem Cell Factor
SCO: Sertoli Cell-Only
SPCs: Spermatogonial Progenitor Cells
SSCs: Spermatogonial Stem Cells
TESE: Testicular Sperm Extraction
VPA: Valproic Acid
VSELs: Very Small Embryonic- Like Cells
